# Activation of SF_5_CF_3_ by the *N*-Heterocyclic Carbene SIMes

**DOI:** 10.3390/molecules28186693

**Published:** 2023-09-19

**Authors:** Domenique Herbstritt, Pooja Tomar, Thomas Braun

**Affiliations:** Department of Chemistry, Humboldt–Universität zu Berlin, Brook-Taylor-Str. 2, 12489 Berlin, Germany

**Keywords:** carbenes, defluorination, fluorination, trifluoromethylation, sulfur fluorides

## Abstract

The greenhouse gas SF_5_CF_3_ was photochemically activated with SIMes (1,3-Bis(2,4,6-trimethylphenyl)-4,5-dihydroimidazol-2-ylidene) to give 1,3-dimesityl-2,2-difluoroimidazolidine (SIMesF_2_), and 1,3-dimesitylimidazolidine-2-sulfide, as well as the trifluoromethylated carbene derivative 1,3-dimesityl-2-fluoro-2-trifluoromethylimidazolidine. CF_3_ radicals, as well as SF_4_, serve presumably as intermediates of the conversions. In addition, the photochemical activation of SF_5_CF_3_ was performed in the presence of triphenylphosphine. The formation of triphenyldifluorophosphorane and triphenylphosphine sulfide was observed.

## 1. Introduction

The greenhouse gases SF_5_CF_3_ and SF_6_ are both chemically highly inert and have a long atmospheric lifetime [1,2,3]. Whereas the activation of SF_6_ has been well established in the last decade [4,5,6,7,8,9,10,11,12,13,14,15,16,17], the studies on SF_5_CF_3_ are rare. Dresdner et al. published the reaction of SF_5_CF_3_ with perfluoropropylene at temperatures of 425 °C–513 °C to give perfluoroethane and SF_4_ [18,19,20]. Huang et al. were able to decompose the greenhouse gases SF_6_ and SF_5_CF_3_ via photolysis in the presence of propene at 184.9 nm [21]. Another SF_5_CF_3_ activation was performed at the rhodium hydrido complex [{Rh(µ-H)(dippp)}_2_] (dippp = 1,3-bis(diisopropylphosphanyl)propane) to yield a binuclear rhodium compound bearing a bridging SCF_3_ ligand [16]. We previously reported on a photocatalytic reduction of SF_5_CF_3_ using [Ir(dtbbpy)(ppy)_2_]PF_6_ (4,4′-di-*tert*-butyl-2,2′-dipyridyl, ppy = 2-phenylpyridine) as the photocatalyst and NEt_3_ as the reductant. The generation of a CF_3_ radical led to the development of a process for the trifluoromethylation of aromatics [22]. With regard to the activation of SF_6_, the *N*-heterocyclic carbene SIMes was used to achieve a photolytic activation at 311 nm, yielding 1,3-dimesityl-2,2-difluoroimidazolidin (SIMesF_2_, **2**) and 1,3-dimesitylimidazolidine-2-sulfide. It was also shown that alcohols can be subsequently fluorinated in situ [15]. Rotering et al. demonstrated that triphenylphosphine can be utilized to activate SF_6_ under irradiation to yield Ph_3_PF_2_ and Ph_3_P=S in a ratio of 3:1. The latter mixture was used in situ for the deoxyfluorination of carboxylic acids [23]. For both processes, SF_6_ was presumably initially reduced to give SF_6_^−^. The latter can generally transform into an SF_5_ radical and a fluoride, or into SF_5_^−^ and a fluorine radical [17,24,25]. SF_5_CF_3_, however, produces CF_3_ radicals and the SF_5_^−^ anion after reduction [26,27]. In this paper, we report on the thermal and photochemical activation of SF_5_CF_3_ by the *N*-heterocyclic carbene SIMes to result in fluorinated and trifluoromethylated heterocycles [28]. The photochemical activation process of SF_5_CF_3_ using triphenylphosphine was studied for comparison.

## 2. Results

### 2.1. Thermal Activation of SF_5_CF_3_ with SIMes

**Scheme 1:**
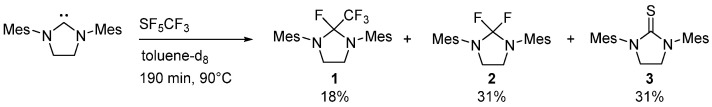
Activation of SF_5_CF_3_ at 90 °C in toluene-*d*_8_.

**Figure 1:**
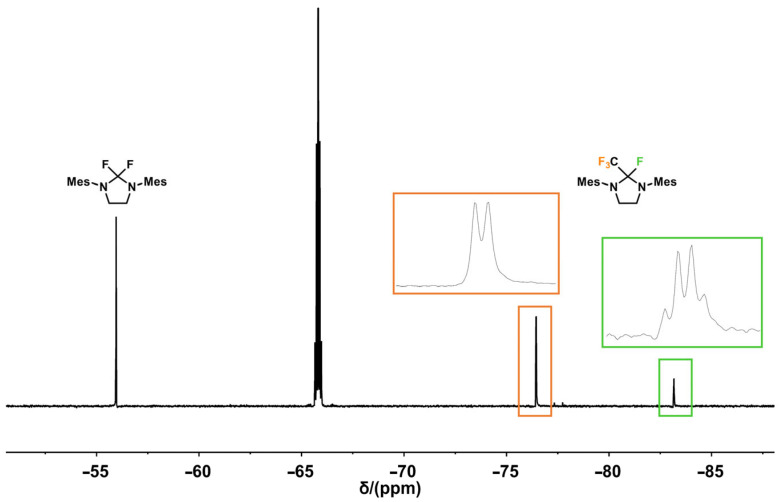
^19^F NMR spectrum for SIMesF_2_ **2** and **3**.

Heating a 1:1 mixture of SIMes and SF_5_CF_3_ at 90 °C for 190 min in toluene-*d*_8_ led to the formation of 1,3-dimesityl-2-fluoro-2-trifluoromethylimidazolidine (**1**), SIMesF_2_ (**2**) and 1,3-dimesitylimidazolidine-2-sulfide (**3**), with NMR yields of 18%, 31%, and 31% based on the amount of SF_5_CF_3_ (Scheme 1). The ^19^F NMR spectrum (Figure 1) of the mixture shows a signal at *δ* = −55.8 ppm for SIMesF_2_ (**2**), which is consistent with the literature [15,28,29], as well as a doublet at *δ* = −76.3 with a coupling constant ^3^J_FF_ of 4.2 Hz, and a quartet at *δ* = −83.1 with a coupling constant ^3^J_FF_ of 4.4 Hz for compound **1**. The formation of **3** was further confirmed through comparing the ^1^H NMR spectrum with the data reported in the literature [15]. It was noted that traces of trifluoromethane were also observed according to the ^19^F NMR spectrum. Heating the sample further for one hour led to a decrease in the amount of **1** and SIMesF_2_ (**2**) and, instead, more trifluoromethane and trifluoromethane-d_1_ were observed. The latter can be formed due to the reaction of an intermediate CF_3_ radical (see below) via hydrogen or deuterium atom transfer. Attempts to achieve a similar transformation in benzene gave the considerably lower amounts of **1**, **2,** and **3**, possibly due to the lower reaction temperature, which was limited by the boiling point of benzene.

### 2.2. Photolytic Activation of SF_5_CF_3_ with SIMes

**Scheme 2:**
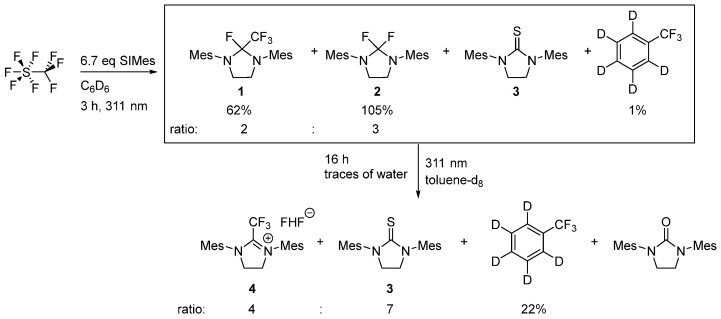
The irradiation of SIMes and SF_5_CF_3_ in C_6_D_6_ at 311 nm (the NMR yields are based on the amount of SF_5_CF_3_).

When a mixture of SF_5_CF_3_ and 6.7 equivalents of SIMes was irradiated at 311 nm in benzene-*d*_6_ for 3 h, the formation of 62% of **1**, and 105% of **2** and **3** was observed, as well as 1% of α,α,α-trifluorotoluene-d_5_ (Scheme 2). All yields are NMR yields based on the amount of SF_5_CF_3._ Further irradiation for 16 h led to the hydrolysis of **2** by adventitious water, to give a urea derivative and HF. The generation of **4** could then be due to the presence of HF. Compound **4** and the thiourea product **3** were observed in a ratio of 4:7. In addition, as described below, the trifluoromethylation of C_6_D_6_ will result in the generation of DF. DF can subsequently lead an FDF^−^ derivative of **4**. Compound **4** shows a singlet at −65.68 ppm in the ^19^F NMR spectrum for the CF_3_ group and a broad signal at −169.40 ppm, indicating the presence of an FHF^−^ anion. The presence of the cation was also confirmed via ESI-MS. Independently synthesized 1,3-dimesityl-2-trifluoromethylimidazolinium tetrafluoroborate showed the same signal in the ^19^F NMR spectrum. The formation of α,α,α-trifluorotoluene-d_5_ with a yield of 22% (based on SF_5_CF_3_) was confirmed via ^19^F NMR spectroscopy and GC-MS. The irradiation of a benzene-*d*_6_ solution of SF_5_CF_3_ at 311 nm for 168 h without the presence of SIMes gave α,α,α-trifluorotoluene-d_5_ with a yield of 2% only. With toluene-*d*_8_ as a solvent, the photochemical activation of SF_5_CF_3_ with SIMes led to the formation of CD_3_C_6_D_5_CF_3_, although the reaction is not selective, and small amounts for unknown products can be detected in the ^19^F NMR spectrum. Trifluoromethylation proceeded at the *ortho* (9%, NMR yield based on the consumption of SF_5_CF_3_) and *para* (5%) position of toluene-*d*_8,_ but also at the *meta* position (4%) [22,30,31].

### 2.3. Mechanisms for the Activation of SF_5_CF_3_

**Scheme 3:**
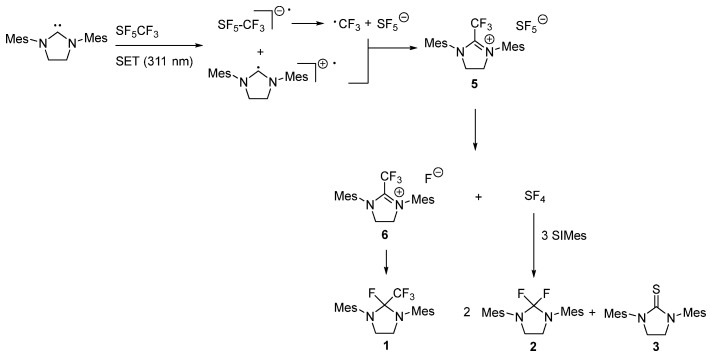
The proposed mechanism for the activation of SF_5_CF_3_ with SIMes in benzene-*d*_6_.

Compound **1** was then investigated regarding its ability to transfer a CF_3_ group to aromatics. Thus, the reaction mixture of **1**, SIMesF_2_ (**2**) and **3** in C_6_D_6_, obtained for the thermal SF_5_CF_3_ activation, (Scheme 1) was degassed under a vacuum to remove any excess of SF_5_CF_3_ and irradiated afterwards at 311 nm. No formation of α,α,α-trifluorotoluene-d_5_ was observed. This suggests that **1** is not capable of transferring a CF_3_ group to aromatics. However, as mentioned above, the formation of compound **1** resembles a process known in the literature for the photochemical activation of SF_6_ by *N*-heterocyclic carbenes, which is initiated by an electron transfer [15,28]. Thus, a SET (single-electron transfer) after carbene excitation to SF_5_CF_3_ can be proposed to give a carbene radical cation and SF_5_CF_3_^−^ radical anion (Scheme 3). The formation of an *N*-heterocyclic carbene radical cation as an intermediate has been proposed by Severin et al. in their discussion of the reaction between SIMes and [Ph_3_C][B(C_6_F_5_)_4_] to yield [SIMes-C_6_H_5_–CPh_2_]^+^ at −40 °C [32,33]. The SF_5_CF_3_^−^ radical anion will then decompose to give SF_5_^−^ and a CF_3_ radical. The latter transformation is consistent with low-temperature electron attachment experiments [27,34,35]. The formed CF_3_ radical recombines with the SIMes radical cation to form **5** bearing an SF_5_^−^ anion. The SF_5_^−^ anion can convert into SF_4_ and **6** [24,36,37]. Compound **6** then reacts to give the observed compound **1** via the nucleophilic attack of the fluoride. SF_4_ reacts further, yielding SIMesF_2_ (**2**) and the thiourea derivative **3**, as was shown in independent experiments [15]. For the thermal activation of SF_5_CF_3_, a comparable transformation can be imagined, although electron transfer can be hampered by a kinetic barrier. In this regard, an incipient transition state or pre-interaction of the nucleophilic carbene with SF_5_CF_3_ seems to be conceivable [8,23,38,39]. It should be noted that an ion flow tube study shows that OH^−^ reacts with SF_5_CF_3_, yielding CF_3_OH and SF_5_^−^ [40]. As mentioned above, DF can be formed in association with the photochemical trifluoromethylation of the aromatic compounds. For this process, initially a cyclohexadienyl radical via reaction with a CF_3_ radical with C_6_D_6_ might be generated [22,30,41,42,43]. The cyclohexadienyl radical can then transfer an electron to the SIMes radical cation, giving a cyclohexadienyl cation. The latter reacts with a fluoride, which stems from SF_5_^−^, and forms α,α,α-trifluorotoluene-d_5_, as well as DF. 

**Scheme 4:**
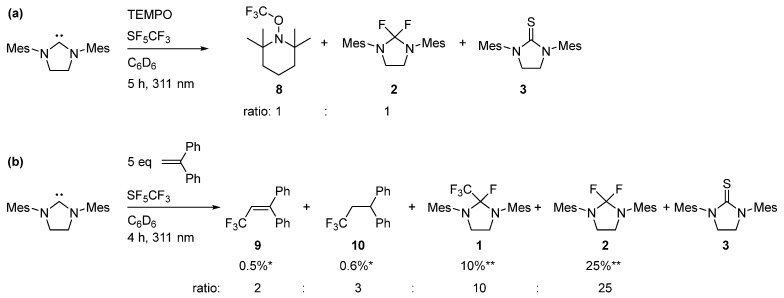
Experiments to trap CF_3_ radicals (**a**) via the addition of TEMPO, and (**b**) via the addition of 1,1-diphenylethylene (* NMR yield based on the amount of 1,1-diphenylethylene, ** NMR yield based on the amount of SIMes).

To confirm the presence of radical intermediates, TEMPO (2,2,6,6-tetramethylpiperidinyloxy) was added to a mixture of SIMes and SF_5_CF_3_ in C_6_D_6_. After 5 h of irradiation at 311 nm, signals for SIMesF_2_ (**2**) and TEMPO-CF_3_ (**8**) [44] in a ratio of 1:1 were observed in the ^19^F NMR spectrum (Scheme 4). The presence of the thiourea derivative **3** was confirmed via ^1^H NMR spectroscopy, as well as via GC-MS. It should be noted that the addition of TEMPO to the reaction of SF_5_CF_3_ with SIMes under non-photolytic conditions did not show the formation of TEMPO-CF_3_, which indicates that no CF_3_ radicals were formed. 

Furthermore, 1,1-diphenylethylene was used as an additional trapping reagent for the CF_3_ radical. After irradiation at 311 nm for 4 h, the trifluoromethylated olefin **9** (0.5% based on the amount of 1,1-diphenylethylene), and the trifluoroalkane **10** (0.6% based on the amount of 1,1-diphenylethylene) were observed in a ratio of 2:3, as well as SIMesF_2_ **2** (25% based on the amount of SIMes), **1** (10% based on the amount of SIMes), and the thiourea derivative **3**, among traces of other compounds, such as trifluoromethane (Scheme 4). Mechanistically, the CF_3_ radical reacts with 1,1-diphenylethylene, and a trifluoromethylbenzyl radical is formed. Two molecules of the latter can generate the olefin **9** and the alkane **10** via hydrogen atom transfer. The formation of trifluoromethane can be explained by HAT from the trifluoromethylbenzyl radical to also yield **9**. 

### 2.4. Activation of SF_5_CF_3_ with Triphenylphosphine

**Scheme 5:**
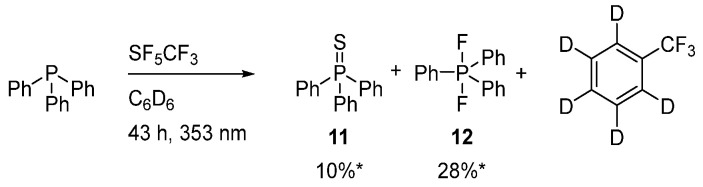
The reduction of SF_5_CF_3_ with triphenylphosphine (* based on the amount of PPh_3_).

The described reactivity of SIMes was compared with that of PPh_3_. Thus, the photolysis of PPh_3_ and SF_5_CF_3_ at 353 nm led to the formation of **11** with a yield of 10%, and **12** with a yield of 28% (based on the amount of PPh_3_, see Scheme 5). α,α,α-Trifluorotoluene-d_5_ was formed, as well. Additionally, traces of F_3_PPh_2_, Ph_2_PF_4_^−^, and O=PPh_2_F were observed, according to the ^19^F NMR spectra [45]. In contrast to the described reactivity with SIMes, the generation of a phosphorane containing a CF_3_ group was not observed. The products were identified via ^31^P NMR, ^19^F NMR spectroscopy, as well as ESI-MS, and the data are consistent with compounds known in the literature [23]. Irradiation at a wavelength of 375 nm led to the formation of only small amounts of phosphorane **12,** and only traces of α,α,α-trifluorotoluene-d_5_. No thermal activation of SF_5_CF_3_ in toluene-*d*_8_ could be achieved via heating the reaction solution at 100 °C for 9 h. Notably, Buß et al. reported on the thermal activation of SF_6_ using strongly basic phosphines [8], but could not observe the thermal activation of SF_6_ with PPh_3_, due to the lower nucleophilicity of the latter [23].

**Scheme 6:**
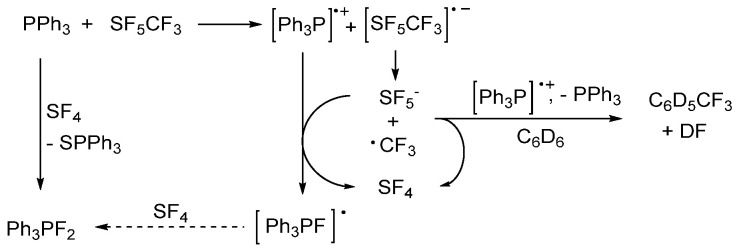
The proposed mechanism for the activation of SF_5_CF_3_ with triphenylphosphine.

A possible mechanism involves SET from the phosphine to the SF_5_CF_3_, resulting in the formation of a SF_5_CF_3_^−^ radical anion and a PPh_3_^+^ radical cation (Scheme 6). The SF_5_CF_3_ radical anion then decomposes to give a CF_3_ radical and a SF_5_^−^ anion [27,34,35]. The latter can either decompose to fluoride and SF_4_, or give a Ph_3_PF radical via reaction with PPh_3_^+^. PPh_3_ reacts with SF_4_, yielding F_2_PPh_3_ and SPPh_3._ The generated Ph_3_PF might become further fluorinated via intermediate sulfur fluorides or SF_4_, to yield PPh_3_F_2._ The CF_3_ radical reacts with the solvent C_6_D_6_, yielding α,α,α-trifluorotoluene-d_5_ and, presumably, DF, possibly via the re-oxidation of a cyclohexadienyl radical cation with PPh_3_^+^, and subsequent deprotonation with fluoride_._ It should be noted that Dielmann et al. also proposed, for the photochemical activation of SF_6_ with triphenylphosphine, a mechanism based on DFT calculations, in which an electron is initially transferred from a π orbital of an arene moiety of PPh_3_ to the delocalized σ* orbital of SF_6_ [23]. 

## 3. Materials and Methods

### 3.1. General Instruments, Methods, and Materials

All reactions were performed under an argon atmosphere, to exclude air and moisture. Chemicals were stored in an argon-filled glass apparatus, using the standard Schlenk-technique. SIMes was synthesized from 1,3-Bis(2,4,6-trimethylphenyl)-4,5-dihydroimidazol-1-ium tetrafluoroborate, KO*t*Bu, and NaH, all of which were heated at night under a vacuum prior to use. TEMPO, SF_5_CF_3_, and PPh_3_ were purchased from commercial sources, and used without further purification. 1,1-Diphenylethylene was stored over a molecular sieve (3 Å) before use. Toluene-*d*_8_, C_6_D_6_, and THF-*d*_8_ were stored over Solvona^®^. All solvents were distilled and degassed prior to use, and stored under argon over molecular sieves (3 Å). As the light source, an LED lamp with a peak wavelength of 375 nm from Innotas Produktions GmbH (Zittau, Germany) was used, as well as a photo Multirays reactor (Helios Italquartz, Cambiago, Italy) equipped with ten light sources (15 W), with an emission maximum of 311 nm or 353 nm. The NMR spectra were recorded at room temperature with a Bruker AV III 300 or Bruker DPX 300 spectrometer (Ettlingen, Germany). The chemical shifts in the ^1^H and ^13^C{^1^H} NMR spectra were calibrated to the residual solvent signal of the deuterated solvents. The ^1^H NMR spectra were referenced as C_6_D_5_H: *δ* = 7.16 ppm; toluene-d_7_: *δ* = 6.97 ppm; CHD_2_CN: *δ* = 1.94 ppm, and CHDCl_2_: *δ* = 5.32 ppm. The ^13^C{^1^H} NMR spectra were referenced as C_6_D_6_: *δ* = 128.06 ppm; toluene-*d*_8_: *δ* = 20.43 ppm; CD_3_CN: *δ* = 1.32 ppm and CD_2_Cl_2_: *δ* = 53.84 ppm. The ^19^F NMR spectra were referenced externally to CFCl_3_ at *δ* = 0.0 ppm. As an internal standard for the quantification, 1-fluoropentane was used with *δ* of −217.6 ppm in the ^19^F NMR spectrum. GC–MS measurements were conducted using an Agilent 6890N gas chromatograph with a capillary column (Agilent 19091S-433 Hewlett-Packard 5 MS: 30 m length, 0.25 mm inside diameter, 0.25 μm film thickness) and an Agilent 5973 Network mass selective detector. Helium (0.74 bar, 1.2 mL/min, 40 cm/s) was used as the carrier gas. The electron impact ionization was carried out with an ionization voltage of 70 eV. Mass spectra were measured with a Micromass Q-Tof-2 instrument, with a Linden LIFDI source (Linden CMS GmbH, Weyhe, Germany). ESI-MS spectra were recorded using an ADVION EXPRESSION CMS spectrometer, as an eluent CD_3_CN was used, and the sample was directly injected into the instruments. The data were analyzed using ADVION DATA EXPRESS Version 6.0.11.3. Caution: in some experiments, traces of HF were generated. Immediate access to procedures in case of contact with HF-containing solutions must be available.

### 3.2. Activation of SF_5_CF_3_ with SIMes by Heating

A PFA tube was filled with SIMes (0.016 g, 0.0525 mmol) and 1-fluoropentane (6 μL, 0.0525 mmol) as an internal standard. The tube was attached to a steel line, and C_6_D_6_ (0.4 mL) was condensed into the PFA tube. After the solvent was degassed, SF_5_CF_3_ (175 mbar, 0.0525 mmol) was condensed into the PFA tube, which was then flame-sealed under a vacuum. The reaction mixture was heated at 90 °C for 3 h. Compound **1** was detected with a yield of 18%, **2** was detected with a yield of 31%, and **3** was detected with a yield of 31%. All yields are NMR yields (internal standard: 1-fluoropentane) based on SF_5_CF_3_.

NMR data for 1,3-dimesityl-2-fluoro-2-trifluoromethylimidazolidine **1**: ^19^F NMR (282.4 MHz, tol-*d*_8_): *δ* = −76.3 (d, 3F, ^3^*J*_FF_ = 3.8 Hz, CF_3_), −83.1 (q, 1 F, ^3^*J*_FF_ = 3.8 Hz, F) ppm.

NMR data for 1,3-dimesityl-2,2-difluoroimidazolidin **2**: ^19^F NMR (282.4 MHz, tol-*d*_8_): *δ* = −55.8 (s) ppm. The obtained NMR data are consistent with those in the literature [15].

Analytical data for 1,3-dimesitylimidazolidine-2-sulfide **3**: ^1^H NMR (300.1 MHz, tol-*d*_8_): δ = 2.09 (m, 6 H, *p*-CH_3_), 2.22 (m, 12 H, *o*-CH_3_), 3.21 (s, 4H, NCH_2_CH_2_N), 6.71 (s, 4H, H_Ar_) ppm., GC-MS (tol-*d*_8_): calculated (*m*/*z*) for [3]: 338.18 experimental (*m*/*z*) for [3]: 338. The obtained NMR data are consistent with those in the literature [15].

Reaction products **1**, **2**, and **3** were degassed (freed of SF_5_CF_3_) and irradiated at 311 nm in the photochemical reactor at 311 nm. After irradiation for 16 h, no trifluoromethylated solvent was observed.

### 3.3. Photochemical Activation of SF_5_CF_3_ with SIMes

A PFA tube was filled with SIMes (0.032 g, 0.105 mmol) and 1-fluoropentane (6 μL, 0.0525 mmol) as an internal standard. The tube was attached to a steel line, and C_6_D_6_ (0.4 mL) was condensed on top. After the solvent was degassed, SF_5_CF_3_ (87 mbar, 0.026 mmol, 1 eq) was condensed into the solution, and the PFA tube was flame-sealed under a vacuum. The reaction mixture was irradiated in a UV reactor (311 nm) at room temperature for 16 h.

Analytical data for **4**: ^19^F NMR (282.4 MHz, C_6_D_6_): *δ* = −170.1 (br s, 2H), −65.6 (s, 3F) ppm., ESI-MS (CD_3_CN): calculated (*m*/*z*) for [4]^+^: 375.2, experimental (*m*/*z*) for [4]^+^: 375.3.

Analytical data for α,α,α-trifluorotoluene-d_5_: ^19^F NMR (282.4 MHz, C_6_D_6_): *δ* = −62.4 ppm., GC-MS (C_6_D_6_): calculated (*m*/*z*) for [α,α,α-trifluorotoluene-d_5_]: 151, experimental (*m*/*z*) for [α,α,α-trifluorotoluene-d_5_]: 151. The obtained NMR data are consistent with those in the literature [22].

### 3.4. Experiments to Trap Radicals

#### 3.4.1. Addition of TEMPO to Reaction Mixture

A PFA tube was filled with SIMes (0.015 g, 0.05 mmol) and TEMPO (0.034 g, 0.22 mmol, 4.4 eq). The tube was attached to a steel line, and C_6_D_6_ (0.4 mL) was condensed on top. After the solvent was degassed, SF_5_CF_3_ (300 mbar, 0.09 mmol, 1.8 eq) was condensed into the solution, and the PFA tube was flame-sealed under a vacuum. The reaction mixture was irradiated in a UV reactor (311 nm) at room temperature for 12 h. TEMPO-CF_3_, SIMesF_2_, and compound **1** (1,3-Bis(2,4,6-trimethylphenyl)-imidazolidin-2-sulfide) were observed via ^19^F and ^1^H NMR spectroscopy and GC-MS.

Analytical data for **8**: ^19^F NMR (282.4 MHz, C_6_D_6_): *δ* = −56.5 ppm. The obtained NMR data are consistent with those in the literature [15,22].

#### 3.4.2. Addition of 1,1-Diphenylethylene

A PFA tube was filled with SIMes (0.016 g, 0.0525 mmol) and 1,1-diphenylethylene (46 µL, 0.263 mmol, 5 eq). The tube was attached to a steel line, and C_6_D_6_ (0.4 mL) was condensed on top. After the solvent was degassed, SF_5_CF_3_ (175 mbar, 0.0525 mmol, 1 eq) was condensed into the solution, and the PFA tube was flame-sealed under a vacuum. The reaction mixture was irradiated in a UV reactor (311 nm) at room temperature for 3 h. Compounds **9** and **10** were observed via ^19^F NMR spectroscopy with a 0.5% and 0.6% NMR yield (compared to 1,1-diphenylethylene), and via GC-MS; SIMesF_2_ **2** was observed via ^19^F NMR spectroscopy with 25% (NMR yield, based on the amount of SIMes), 1,3-dimesityl-2-fluoro-2-trifluoromethylimidazolidine **1** was observed with a yield of 10% (NMR yield, compared to the amount of SIMes), and a signal for compound **3** was observed via ^1^H NMR spectra and GC-MS.

Analytical data for **9**: ^19^F NMR (282.4 MHz, C_6_D_6_): *δ* = −55.6 (t, ^3^*J*_FF_ = 10.2 Hz) ppm, GC-MS (C_6_D_6_): calculated (*m*/*z*) for [9]: 248.08, experimental (*m*/*z*) for [9]: 248. The obtained NMR data are consistent with those in the literature [46,47].

Analytical data for **10**: ^19^F NMR (282.4 MHz, C_6_D_6_): *δ* = −63.3 (t, ^3^*J*_FH_ = 10.2 Hz) ppm., GC-MS (C_6_D_6_): calculated (*m*/*z*) for [10]: 250.10, experimental (*m*/*z*) for [10]: 250. The obtained NMR data are consistent with those in the literature [46,48].

### 3.5. Activation of SF_5_CF_3_ with PPh_3_

A Young flask was filled with PPh_3_ (0.5 g, 1.9 mmol) and 1-fluoropentane (100 μL, 0.875 mmol) as an internal standard. The mixture was dissolved in C_6_D_6_ (20 mL). The Young flask was attached to a steel line. After the solvent was degassed, the flask was filled with SF_5_CF_3_ (1.3 bar). The reaction mixture was irradiated in a UV reactor (353 nm) at room temperature for 43 h. Compounds **11** and **12** were observed via ^19^F and ^31^P NMR spectroscopy. A signal for α,α,α-trifluorotoluene-d_5_ was observed (0.065 mmol) via ^19^F NMR spectroscopy.

Analytical data for **11**: ^31^P NMR (121.5 MHz, C_6_D_6_): *δ* = 42.28 (s)., ESI-MS (CD_3_CN): calculated (*m*/*z*) for [11 + 2Na^+^ + H^−^]^+^: 341.05, experimental (*m*/*z*) for [11 + 2Na^+^ + H^−^]^+^: 341.05. The obtained NMR data are consistent with those in the literature [13,23].

Analytical data for **12**: ^19^F NMR (282.4 MHz, C_6_D_6_): *δ* = −39.0 (d, ^1^*J*_FP_ = 664.44 Hz) ppm., ^31^P NMR (121.5 MHz, C_6_D_6_): *δ* = −55.21 (t, ^1^*J*_FP_ = 663.42 Hz), ESI-MS (CD_3_CN): calculated (*m*/*z*) for [13 + K]^+^: 339.05, experimental (*m*/*z*) for [13 + K]^+^: 339.3. The obtained NMR data are consistent with those in the literature [13,23].

## 4. Conclusions

In conclusions, reaction routes for the activation of the greenhouse gas SF_5_CF_3_ with SIMes and PPh_3_ were developed. Photochemical processes presumably proceed by an initial electron transfer to the fluorinated substrate, and provide CF_3_ radicals. This is revealed via trapping experiments of a CF_3_ radical, and also the trifluoromethylation of C_6_D_6_. The studies complement efforts regarding the activation and degradation of fluorinated compounds [49,50,51,52,53,54,55,56].

## Data Availability

Supporting information containing NMR spectra is available.

## Supplementary Materials

The following supporting information can be downloaded at: https://www.mdpi.com/article/10.3390/molecules28186693/s1, Figures S1–S10: Figure S1: ^19^F NMR spectra for 1,3-dimesityl-2-fluoro-2-trifluoromethylimidazolidine (**1**) and 1,3-dimesityl -2,2-difluoroimidazolidin (**2**) in toluene-*d_8_* after heating of SIMes and SF_5_CF_3_ at 90 °C; Figure S2: ^19^F{^1^H} NMR spectra for 1,3-dimesityl-2-fluoro-2-trifluoromethylimidazolidine (**1**) and 1,3-dimesityl -2,2-difluoroimidazolidin (**2**) in toluene-*d_8_* after heating of SIMes and SF_5_CF_3_ at 90 °C; Figure S3: ^19^F^19^F COSY NMR spectrum for 1,3-dimesityl-2-fluoro-2-trifluoromethylimidazolidine (**1**) in toluene-*d_8_* after heating of SIMes and SF_5_CF_3_ at 90 °C; Figure S4: ^19^F NMR spectrum for 1,3-dimesityl-2-fluoro-2-trifluoromethylimidazolidine (**1**) and SIMesF_2_ (**2**) in C_6_D_6_ after irradiation of SIMes and SF_5_CF_3_ at 311 nm after 3 h; Figure S5: ^1^H NMR spectrum for compounds **3** and **4** in C_6_D_6_ after irradiation of SIMes and SF_5_CF_3_ at 311 nm after 16 h; Figure S6: ^19^F NMR spectrum for compound **4** in C_6_D_6_ after irradiation of SIMes and SF_5_CF_3_ at 311 nm after 16 h; Figure S7: ^19^F NMR spectrum for compound **8** in C_6_D_6_; Figure S8: ^19^F NMR spectrum for compounds **1**, **2**, **9** and **10** in C_6_D_6_ after irradiation of SIMes, SF_5_CF_3_ and 1,1-diphenylethylene at 311 nm for 3 h; Figure S9: ^19^F NMR spectrum for compound **12** and α,α,α-trifluorotoluene-*d_5_* in C_6_D_6_; Figure S10: ^31^P NMR spectrum for compounds **11** and **12** in C_6_D_6_.
