# Supplementary material for: Activation of SF5CF3 by the N-Heterocyclic Carbene SIMes
Source: Molecules. 2023 Sep 19;28(18):6693. doi: 10.3390/molecules28186693 (PMC10535660; doi:10.3390/molecules28186693)
Supplement: Supplementary file 1 [file molecules-28-06693-s001.zip › molecules-2601267-supplementary.pdf]

Supporting Information

Activation of SF_5CF_3 by the *N*-heterocyclic Carbene SIMes

Domenique Herbstritt[1], Pooja Tomar[1], and Thomas Braun[1]*

Humboldt-Universität zu Berlin, Department of Chemistry, Brook-Taylor-Straße 2, D-12489 Berlin, Germany. E-mail: thomas.braun@chemie.hu-berlin.de

NMR Spectra

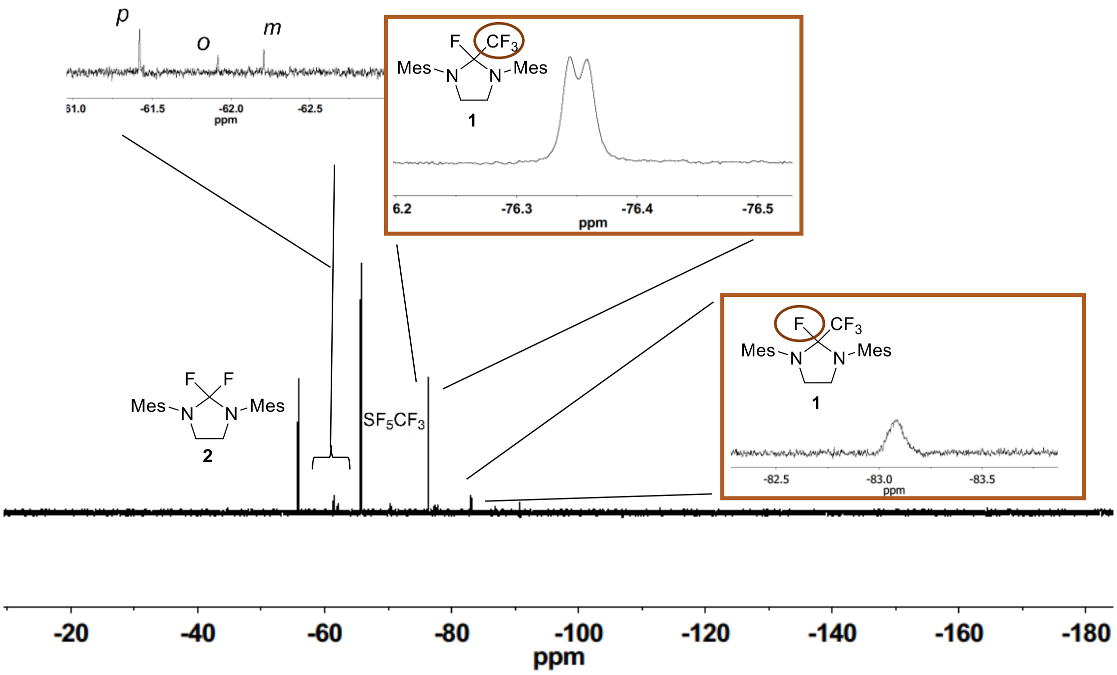

Figure S1: ^{19}F NMR spectra for 1,3-dimesityl-2-fluoro-2-trifluoromethylimidazolidine (**1**) and 1,3-dimesityl -2,2-difluoroimidazolidin (**2**) in toluene-d_8 after heating of SIMes and SF_5CF_3 at 90 °C.

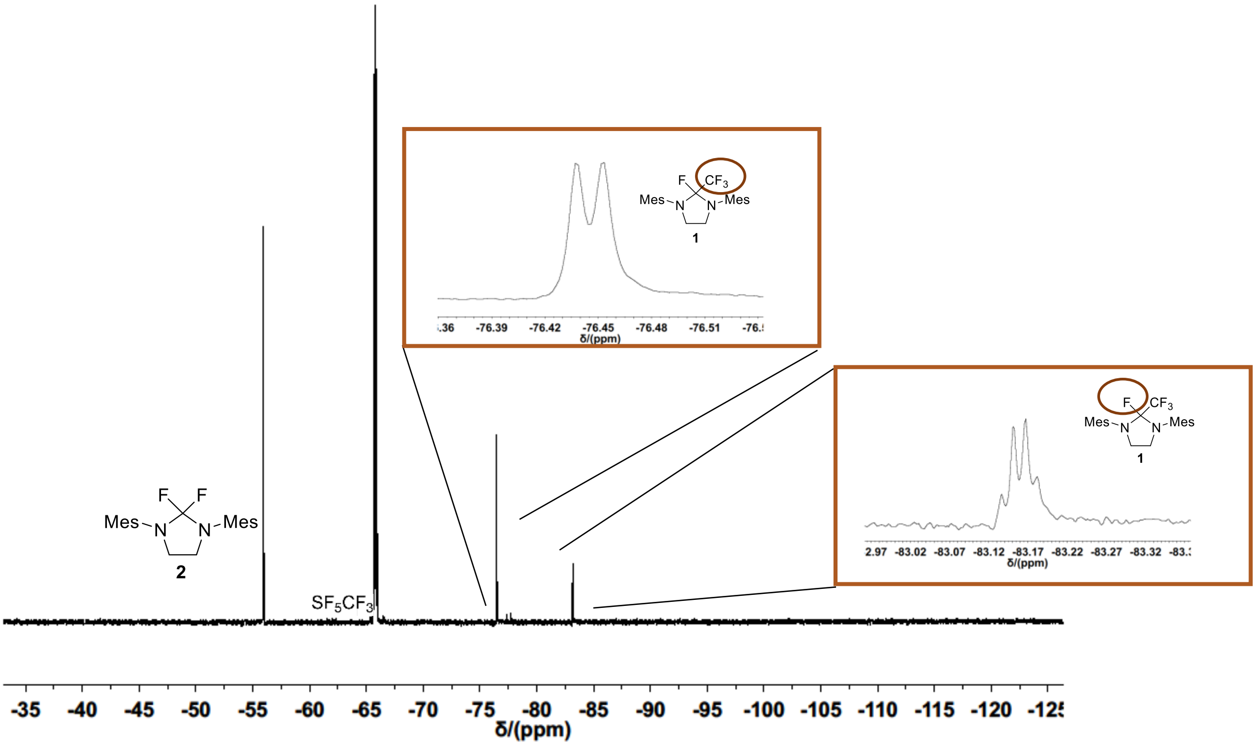

Figure S2: $^{19}F\{^1H\}$ NMR spectra for 1,3-dimesityl-2-fluoro-2-trifluoromethylimidazolidine (**1**) and 1,3-dimesityl -2,2-difluoroimidazolidin (**2**) in toluene-d_8 after heating of SIMes and SF_5CF_3 at 90 °C.

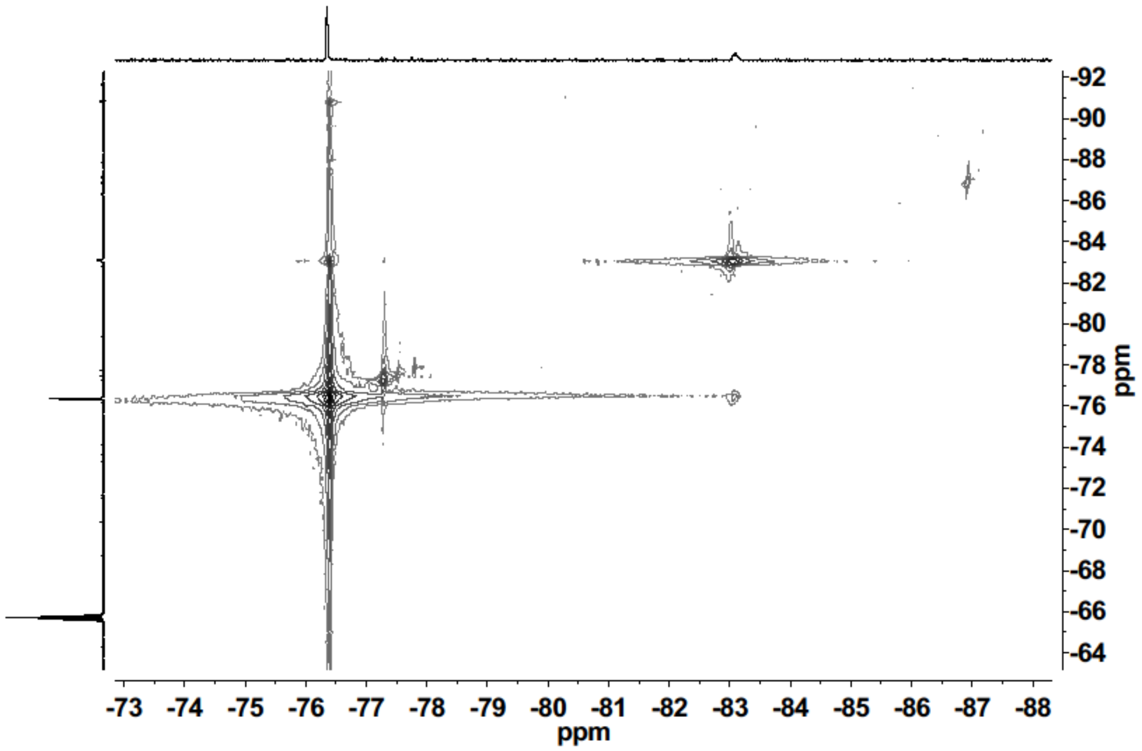

Figure S3: $^{19}F^{19}F$ COSY NMR spectrum for 1,3-dimesityl-2-fluoro-2-trifluoromethylimidazolidine (**1**) in toluene-d_8 after heating of SIMes and SF_5CF_3 at 90 °C.

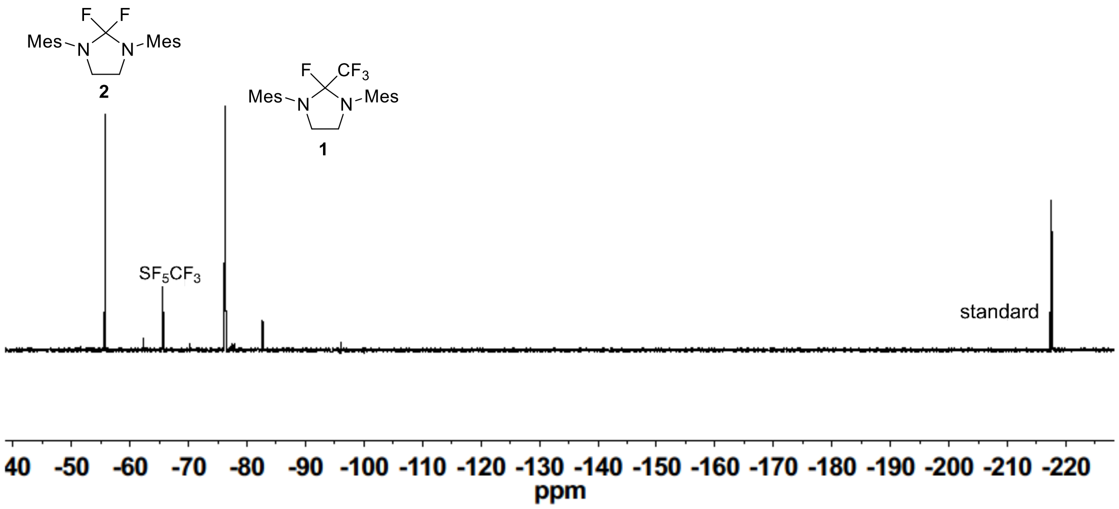

Figure S4: ^{19}F NMR spectrum for 1,3-dimesityl-2-fluoro-2-trifluoromethylimidazolidine (**1**) and SIMesF_2 (**2**) in C_6D_6 after irradiation of SIMes and SF_5CF_3 at 311 nm after 3 h.

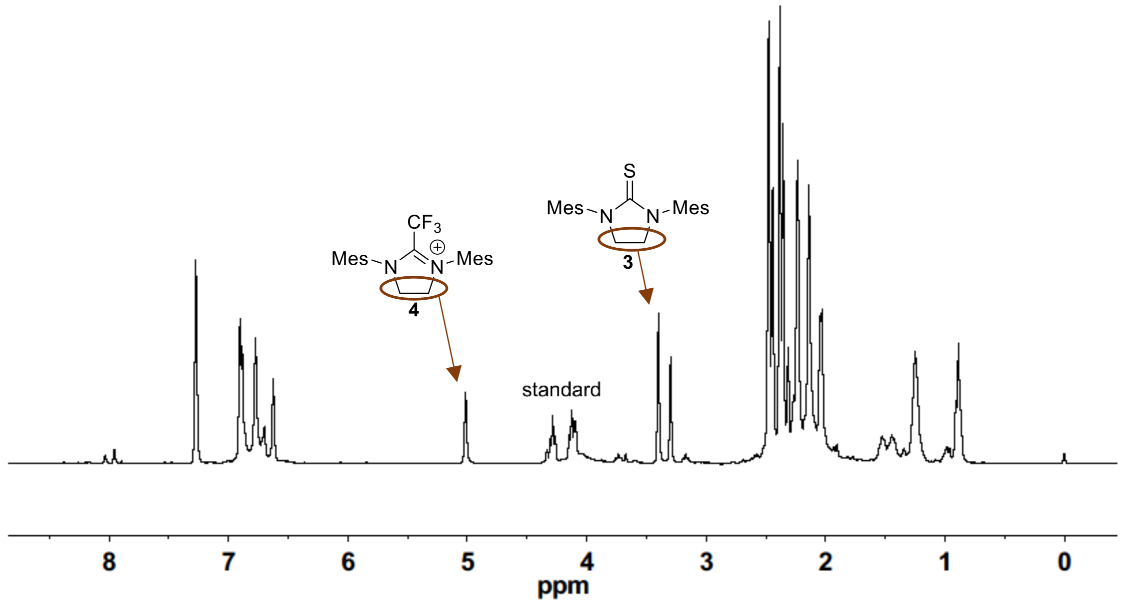

Figure S5: ^{1}H NMR spectrum for compounds **3** and **4** in C_6D_6 after irradiation of SIMes and SF_5CF_3 at 311 nm after 16 h.

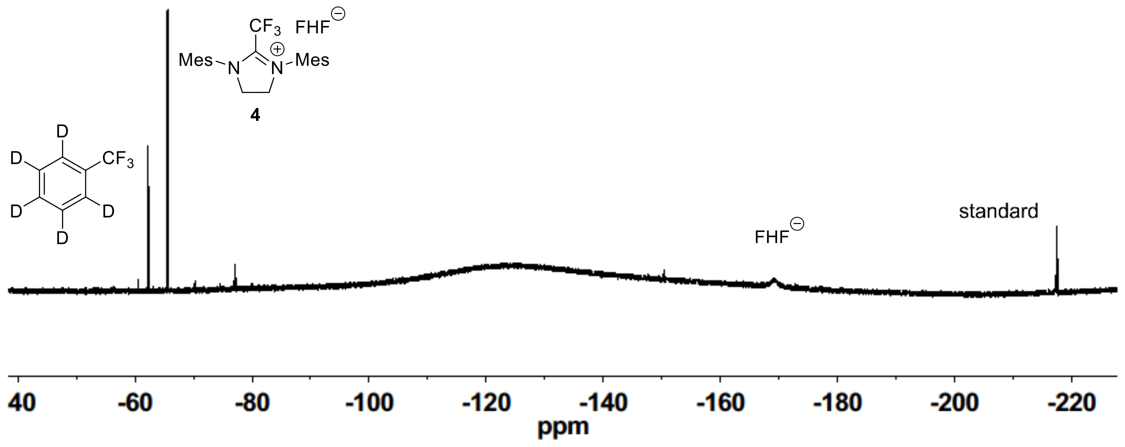

Figure S6: ^{19}F NMR spectrum for compound **4** in C_6D_6 after irradiation of SIMes and SF_5CF_3 at 311 nm after 16 h.

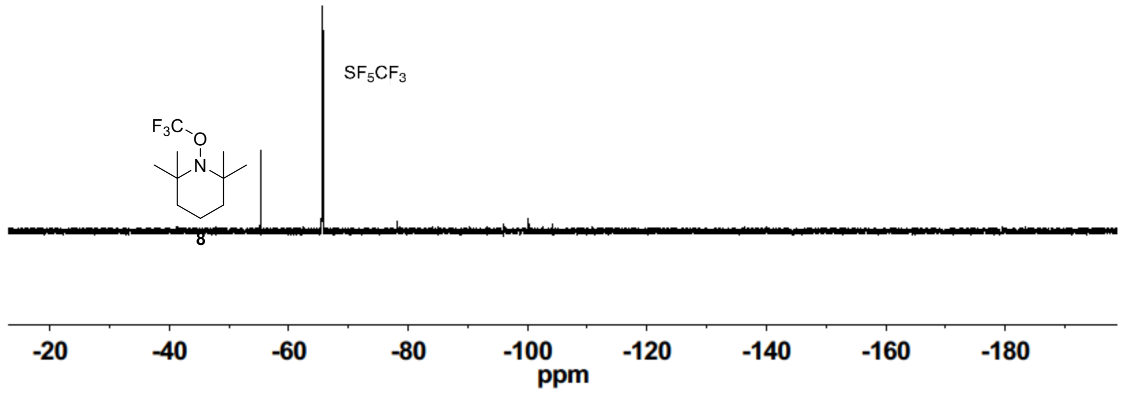

Figure S7: ^{19}F NMR spectrum for compound **8** in C_6D_6.

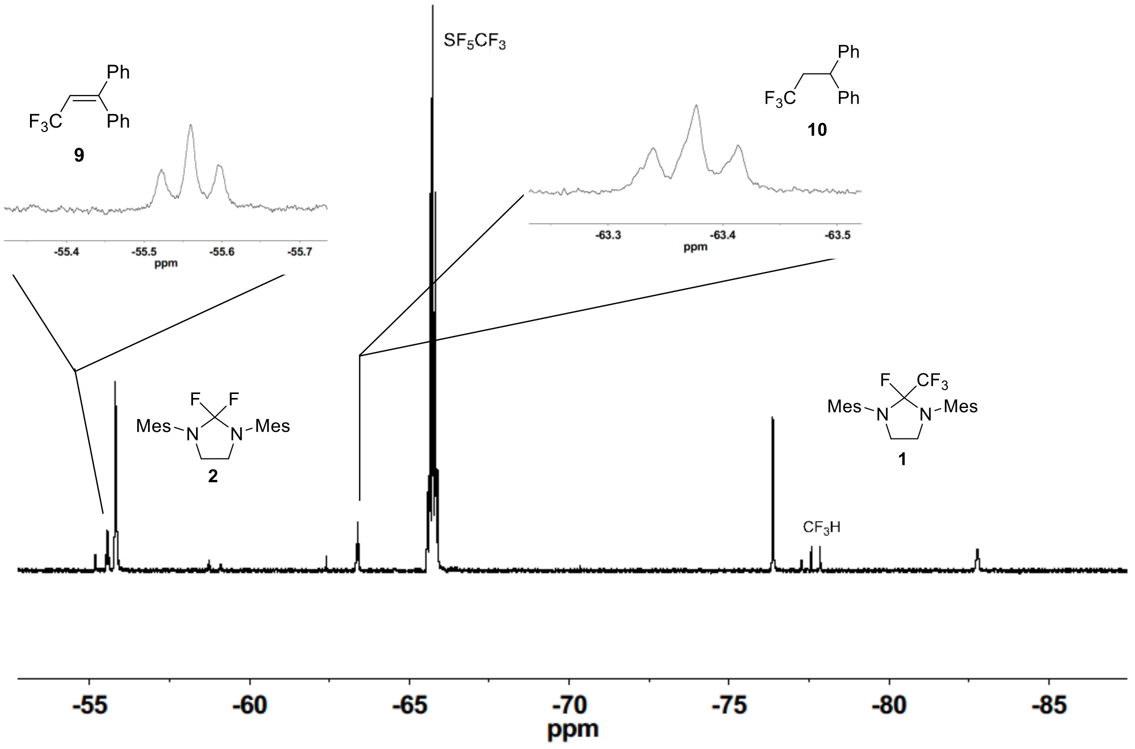

Figure S8: ^{19}F NMR spectrum for compounds **1**, **2**, **9** and **10** in C_6D_6 after irradiation of SIMes, SF_5CF_3 and 1,1-diphenylethylene at 311 nm for 3 h.

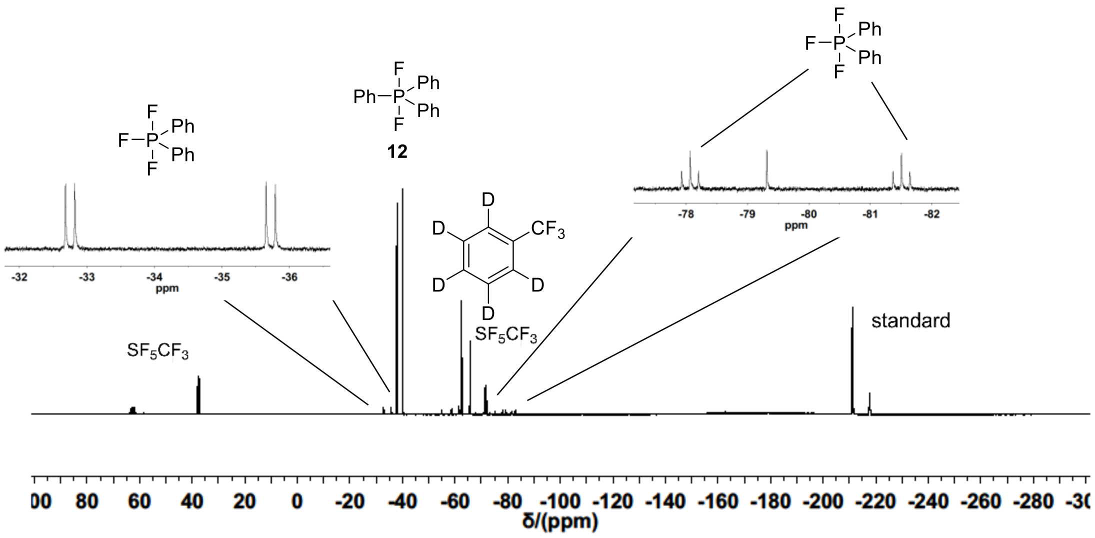

Figure S9: ^{19}F NMR spectrum for compound **12** and α,α,α-trifluorotoluene-d5 in C_6D_6.

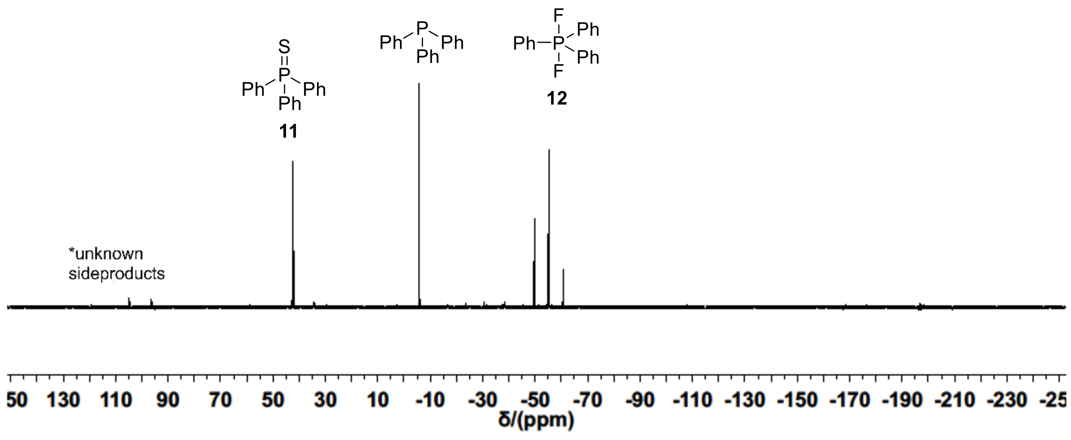

Figure S10: ^{31}P NMR spectrum for compounds **11** and **12** in C_6D_6.
